# ﻿A new genus and two new species of Empoascini (Hemiptera, Cicadellidae, Typhlocybinae) from China

**DOI:** 10.3897/zookeys.1243.147453

**Published:** 2025-07-01

**Authors:** Qing-Ping Yao, Yan Ding, Mao-Fa Yang, Xiao-Fei Yu

**Affiliations:** 1 Institute of Entomology, Guizhou University, Guiyang, 550025, China; 2 Guizhou Key Laboratory for Agricultural Biosecurity, Guiyang 550025, China; 3 College of Tobacco Science, Guizhou University, Guiyang 550025, China

**Keywords:** Checklist, Empoascini, identification key, leafhopper, new genus, new species, pests, taxonomy

## Abstract

A new genus and two new species of Empoascini from China are depicted in detail in this study: *Singulus* Yao & Yu, **gen. nov.**, with a new species *Singulusfurcatus* Yao & Yu, **sp. nov.**, and *Veluclaustrum* Yao & Yang, **sp. nov.** A revised key to males of Chinese species and a checklist of known species of the genus *Velu* are provided.

## ﻿Introduction

Species of the tribe Empoascini (Hemiptera, Cicadellidae, Typhlocybinae) are major pests of agriculture and forestry (Chen, 1979; Qin et al. 2014). Currently, approximately 107 genera and 1396 species of Empoascini have been reported worldwide, while in China, 50 genera and 262 species are known (Xu et al. 2019, 2021a, 2023; Yu et al. 2020; Wang et al. 2021; Dmitriev et al. 2022; Ding et al. 2024).

The genus *Velu* was initially established by Ghauri in 1964, with the type species *V.caricae* Ghauri being sourced from India. Subsequently, Dworakowska (1980) and Sohi and Mann (1986) each added a new species within this genus, and Zhang and Qin (2004a) compiled a comprehensive species list and formulated a taxonomic key for three newly discovered species of *Velu* from China. To date, six species of *Velu* have been reported across the Eastern Oceanic and Palaearctic realms.

In the current study, a new genus with a new species, are described. In addition, one species of Velu is also described from Yunnan, thereby augmenting the existing knowledge of the genus within the Chinese region and contributing to the broader understanding of its global diversity.

## ﻿Material and methods

The morphological terminology adopted in this work was in accordance with the standards set by Southern (1982), Zhang (1990), Dworakowska (1993), Dietrich (2005), and Xu et al. (2021b). External features of specimens were meticulously recorded and the body length, measured from the apex of the vertex to the tip of the wing, was precisely determined using a KEYENCE VHX-6000 digital camera. Additionally, the male genitalia were photographed with high clarity employing a Nikon Eclipse Ni-E compound microscope. All type specimens of the newly described species have been permanently deposited at the Institute of Entomology, Guizhou University, located in Guiyang, China (GUGC).

## ﻿Taxonomy

### ﻿ Singulus

Taxon classificationAnimaliaHemipteraCicadellidae

Yao & Yu gen. nov.

BE80194B-C335-5844-8246-22995CDCB973

https://zoobank.org/DE522CBC-D4B2-46A3-A1A3-D5C347071500

Figs 1–6 , 7–15

#### Type species.

*Singulusfurcatus* Yao & Yu, sp. nov. here designated.

#### Diagnosis.

The new genus is similar to *Alafrasca* Lu & Qin, 2014, *Lumicella* Lu & Qin, 2013, *Schizandrasca* Anufriev, 1972, and *Circinans* Qin & Liu, 2014 in that the CuA veins of hindwings are branched (Fig. 6), but the new genus differs from the above genera by the following characters: the subgenital plate is triangular with a broad base and only one large seta (Figs 8, 10). *Alafrasca* Lu & Qin differs from the new genus in lacking an anal process branching at the apex (Lu and Qin 2014b). *Lumicella* Lu & Qin differs from the new genus in the following aspects: the subgenital plate is not triangular, the paramere is slim, and the apophysis bears a prominent dentifer (Lu et al. 2013). *Schizandrasca* Anufriev, 1972 differs from the new genus in lacking a paired aedeagus processes (Anufriev, 1972a). *Circinans* Qin & Liu differs by the anal process with a broad and extended caudad (Lu and Qin 2014a).

#### Description.

**Figures 1–6.**
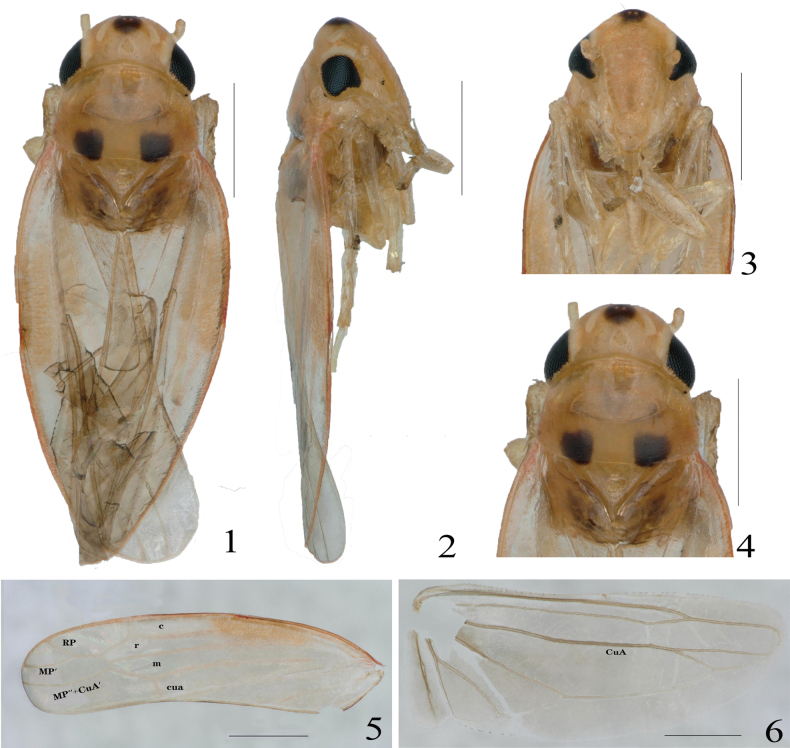
*Singulusfurcatus* Yao & Yu, sp. nov., male holotype. **1.** Habitus, dorsal view; **2.** Habitus, lateral view; **3.** Face, anterior view; **4.** Head and pronotum, dorsal view; **5.** Forewing; **6.** Hindwing. Scale bars: 500 μm.

Body slender (Figs 1–4). Crown nearly as wide as pronotum, middle length of crown less than distance between eyes, a black spot in middle of vertex, with ocelli. Coronal suture present and not reaching anterior margin of crown. Pronotum with anterior margin arcuate, angles on both sides of mesoscutellum are black, scutoscutellar sulcus not reaching both margins (Figs 1, 4). Face larger than wide, elevated in lateral view (Figs 2, 3). Forewing RP and MP′ veins stalked at base, arising from r cell, r cell longest, r cell and m cell subequal in width, 1^st^ apical cell largest, followed by 4^th^ apical, 3^rd^ apical triangular, which is about one-third of wing length (Fig. 5). Hindwing CuA vein branched, and branch point located on or at intersection of CuA and MP′′ veins (Fig. 6).

**Figures 7–15.**
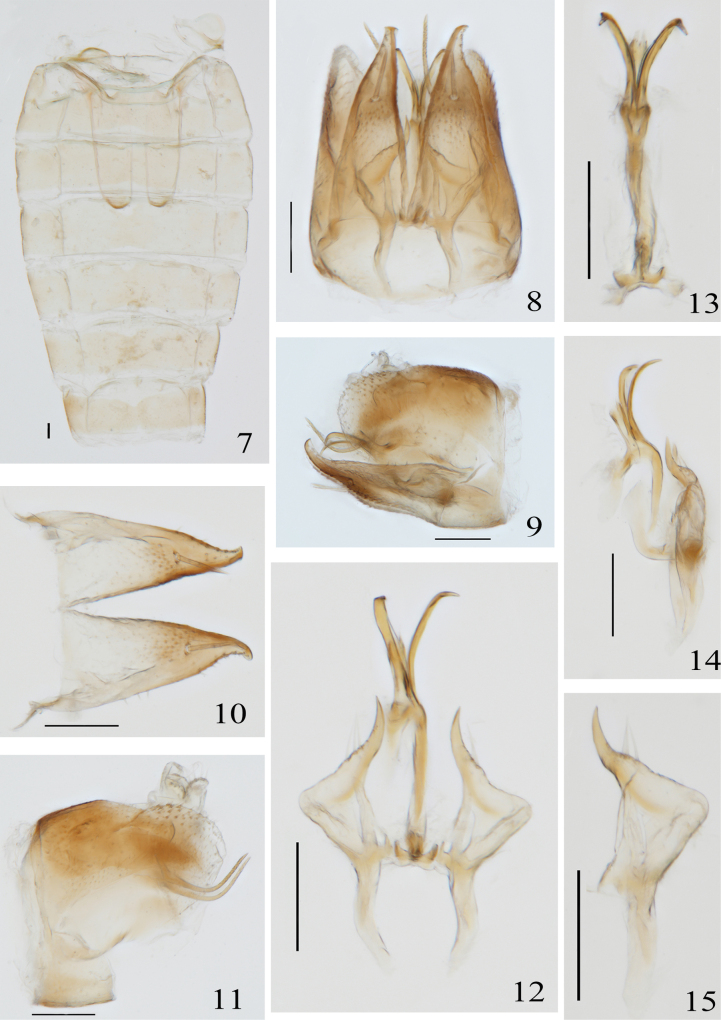
*Singulusfurcatus* Yao & Yu, sp. nov. **7.** Abdominal apodemes; **8.** Male genitalia, ventral view; **9.** Male genitalia, left lateral view; **10.** Subgenital plate; **11.** Pygofer, pygofer appendage and anal tube, lateral view; **12.** Aedeagus, paramere and connective, dorsal view; **13.** Aedeagus and connective, dorsal view; **14.** Aedeagus and connective, left lateral view; **15.** Paramere. Scale bars: 100 μm.

Abdominal apodemes bursts, slender, parallel (Fig. 7). Male genitalia broad, pygofer without process, posterior margin bluntly rounded, with fine setae (Figs 8, 9). Subgenital plate triangular, broad at base, narrowing to tip, with 1 macrosetae (Fig. 10). Apex of paramere is apical, fine setae distributed in subapical, tip without teeth (Figs 12, 15). Aedeagus curved in lateral view, with paired process, without dorsal apodeme, preatrium well developed (Figs 12, 13). Anal process developed, slender, curved toward apex in an arc, exceeding posterior margin of pygofer, proximal with an indentation (Figs 9, 11). Connective not developed (Figs 12, 13).

#### Etymology.

The new genus is named after the subgenital plate of males with only 1 macroseta.

#### Distribution.

Oriental.

### ﻿ Singulus furcatus

Taxon classificationAnimaliaHemipteraCicadellidae

Yao & Yu sp. nov.

B1E66CDB-0061-5776-A015-603F309C9703

https://zoobank.org/96411AF4-525D-4621-9E8A-79762C9AE9A1

Figs 1–6 , 7–15

#### Description.

Body red (Figs 1–4). Anterior margin of crown arcuate, posterior margin concave, a large black spot in middle of anterior margin of crown. Eyes black, ocellus located on top of head in line with antennae, and crown suture conspicuous, not reaching anterior margin of crown. Pronotum red, anterior area with irregular spots, and angles on both sides of scutellum have black spots (Figs 1, 4). Forewings reddish and hindwings translucent (Figs 5, 6).

Male abdominal apodemes extend to middle of 5^th^ abdominal segment, and they are slender and parallel (Fig. 7). Lateral valves of male pygofer side broad at base, narrowed in terminal half, coarsely setae, without ventral appendage (Fig. 9). Anal process developed, extending toward tip, distinctly exceeding posterior margin of pygofer, subterminally with many grooves (Fig. 9). Subgenital plate gradually narrowed basally to tip, with 1 macrosetae and 17 marginal setae (Figs 9, 10). Paramere curved, inflated at middle, pointed at tip and without teeth (Figs 12, 15). Aedeagus curved in lateral view, with short whiskers at apex of shaft and with a pair of protuberances on each side of ventral margin of shaft, which are distinctly curved ventrally beyond apex of shaft, and in dorsal view with shaft protuberances forked (Figs 12, 13, 14). Connective not developed (Figs 12, 13).

#### Material examined.

***Holotype***: • ♂, Mengla, Yunnan, 2015-May-14, coll. Bin Yan. ***Paratypes***: • 2♂, same as holotype; 1♂, Dading Mountain, Guangdong, August 11–13, 2006, coll. Zhong-Hui Zhou.

#### Etymology.

The name of the new species derives from the shaft having a pair of protuberances, which are distinctly forked at the tips when viewed dorsally.

#### Measurement.

Length of male 2.7–2.9 mm.

#### Distribution.

China (Yunnan, Guangdong).

### ﻿Genus Velu

Taxon classificationAnimaliaHemipteraCicadellidae

Ghauri, 1964

141544F5-B248-52B5-B39B-01E4776F113A

Velu Ghauri, 1964e: 467.

#### Type species.

*Velucaricae* Ghauri, 1964.

#### Diagnosis.

**Figures 16–21.**
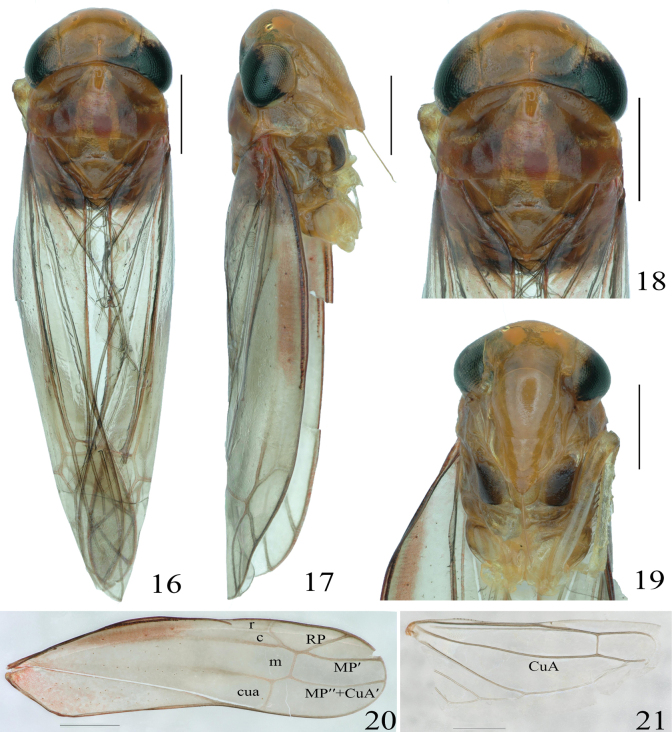
*Veluclaustrum* Yao & Yang, sp. nov., male holotype. **16.** Habitus, dorsal view; **17.** Habitus, lateral view; **18.** Head and pronotum, dorsal view; **19.** Face, anterior view; **20.** Forewing; **21.** Hindwing. Scale bars: 500 μm.

Body robust (Figs 16–19). The anterior margin of the vertex is arc-shaped, and the posterior margin is concave, nearly parallel to each other. The width of the vertex is almost equal to the thorax, and its median length is less than the width between the eyes. Coronal suture is prominent, extending to the middle of vertex, with shallow depressions on both sides; the lateral margin of the pronotum has an inverted trapezoid transverse concavity (Figs 16, 18). Forewing RP and MP’ veins healed at base, and the terminal veins all originate from the m cell, which is wider and longer than the c cell and the r cell. The 3^rd^ apical is triangular, and the submarginal veins of the 2^nd^ apical are nearly parallel (Fig. 20). Hindwing CuA veins are unbranched (Fig. 21).

**Figures 22–31.**
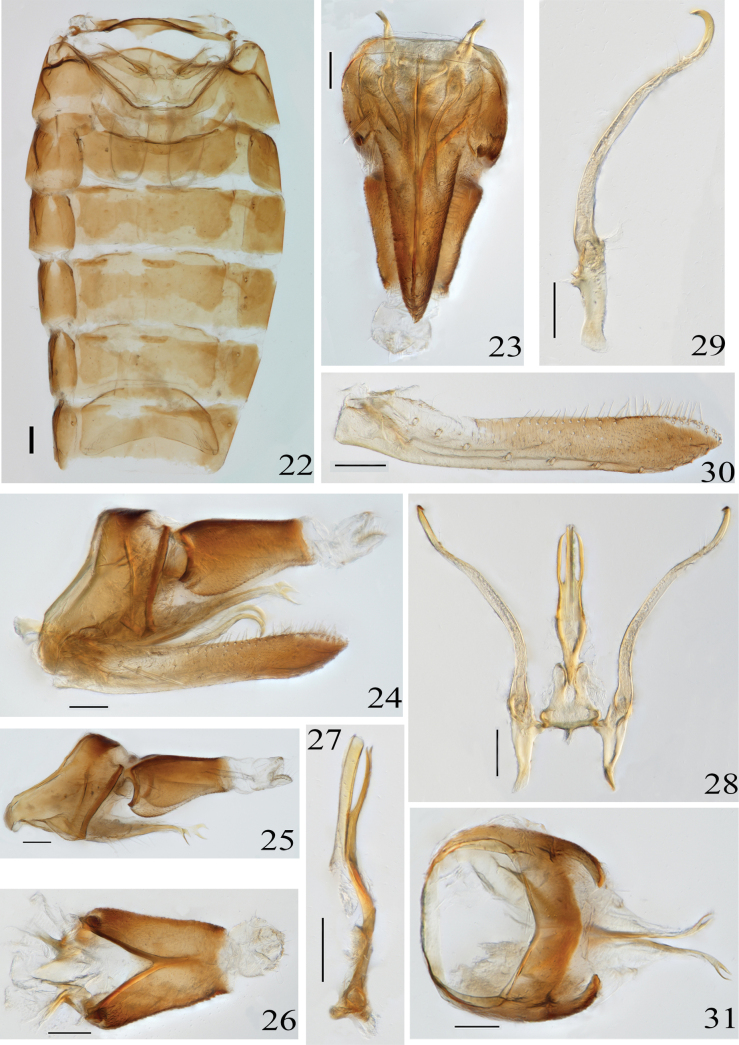
*Veluclaustrum* Yao & Yang, sp. nov. **22.** Abdominal apodemes; **23.** Male genitalia, ventral view; **24.** Male genitalia, left lateral view; **25.** Pygofer, pygofer appendage and anal tube, lateral view; **26.** Anal tube; **27.** Aedeagus, left lateral view; **28.** Aedeagus, paramere and connective, ventral view; **29.** Paramere; **30.** Subgenital plate; **31.** Pygofer and pygofer appendage. Scale bars: 100 μm.

Male abdominal apodemes are well developed and parallel (Fig. 22). Pygofer appendage elongate (Fig. 31). Subgenital plate arranged a row of macrosetae (Fig. 30). Aedeagus has a preatrium, with a process at the base of shaft in ventral view or not (Fig. 27). Paramere slender, curved in a semicircle at the apex, and fine setae are present on the inner margin at the curved portion (Figs 28, 29).

#### Distribution.

Oriental and Palaearctic.

##### ﻿Checklist and distribution of *Velu* Ghauri, 1964

**1. *Veluclaustrum* Yao & Yang, sp. nov.**

**Distribution.** China (Yunnan).

**2. *Velucaricae* Ghauri, 1964**

*Velucaricae* Ghauri, 1964e: 468.

**Distribution.** Pakistan, India (Karnataka, West Bengal).

**3. *Velupruthii* Dworakowska, 1980**

*Velupruthii* Dworakowska, 1980: 155.

**Distribution.** India (Karnataka).

**4. *Veluantelopus* Sohi & Mann, 1986**

*Veluantelopus* Sohi & Mann, 1986: 100.

**Distribution.** India.

**5. *Velufurcatum* Zhang & Qin, 2004**

*Velufurcatum* Zhang & Qin, 2004: 276.

**Distribution.** China (Yunnan).

**6. *Velulongiprojectum* Zhang & Qin, 2004**

*Velulongiprojectum* Zhang & Qin, 2004: 277.

**Distribution.** China (Yunnan).

**7. *Velupleuroprominens* Zhang & Qin, 2004**

*Velupleuroprominens* Zhang & Qin, 2004: 278.

**Distribution.** China (Yunnan).

### ﻿Key to species (males) of *Velu* Ghauri from China

**Table:** 

1	Apex of ventral pygofer appendage unbranched	***V.pleuroprominens***
–	Apex of ventral pygofer appendage branched	**2**
2	Aedeagus with two processes	**3**
–	Aedeagus shaft base only with a forked process	***V.furcatum***
3	The basal processes of aedeagus shaft located on both sides of the shaft, symmetrical, not exceeding shaft length	***V.claustrum* sp. nov.**
–	The basal processes of aedeagus shaft are asymmetrical and unequal in length	***V.longiprojectum***

### ﻿ Velu claustrum

Taxon classificationAnimaliaHemipteraCicadellidae

Yao & Yang sp. nov.

18BC040A-5D62-52FB-A233-C14081AE02E8

https://zoobank.org/876ACE3F-9A03-493A-A415-B7B006AE54AF

Figs 16–21 , 22–31

#### Description.

Whole body orange-yellow (Figs 16–19). Anterior margin of head curved, posterior margin concave, ocelli brown at junction of head and face, eyes light brown, and crown suture conspicuous, reaching to middle of crown. Pronotum with anterior margin arcuate, anterior domain with a transverse dark depression, middle and posterior domains orange, elevated, and intershield grooves brown, not reaching both margins (Figs 16, 18). Face broad, frontclyeal area elevated in lateral view (Figs 17, 19). Forewing RP and MP’ veins heal at base, m-cells wider than and longer than c cells and r cells, 3^rd^ apical triangular, 2^nd^ apical lateral veins subparallel (Fig. 20). Hindwing CuA veins unbranched (Fig. 21).

Male abdominal apodemes extending to 4^th^ abdominal segment (Fig. 22). Pygofer side lateral view basally broad, apex membranous, dorsal margin abruptly narrowed near middle, resulting in large difference in width between terminal and basal pygofer side lateral view, and pygofer appendage long, distinctly exceeding apex of pygofer, apex split (Figs 23, 24, 25, 31). Anal process absent (Fig. 26). Subgenital plate long, base slightly broad, subequal in width in terminal half, with 8 individual macrosetae, 39 long, fine setae (Fig. 30). Paramere long and narrow, hooked and curved in terminal half, with fine setae subterminally (Figs 28, 29). Aedeagus curved in lateral view, with a pair of lateral processes at base of shaft, which are slightly shorter and narrower than shaft, while apex of paramere curved towards shaft in ventral view (Figs 27, 28). Connective base broad, terminal margin deeply concave (Figs 27, 28).

#### Material examined.

***Holotype***: • 1♂, Menghai, Xishuangbanna, Yunnan, 2013-July-14, coll. Ji-Chun Xing. ***Paratypes***: • 1♂, 4♀, same collecting information as holotype.

#### Etymology.

The new species is named after the pair of dark-coloured striped depressions on both margins of the pronotum in specimens examined.

#### Measurement.

Length of males 3.9–4.0 mm, females 4.1–4.5 mm.

#### Remarks.

The new species is similar to *V.pleuroprominens* Zhang & Qin, 2004 in having a well-developed preatrium, slightly shorter than the shaft, and symmetrical lateral processes of the shaft (Fig. 28). The latter species is different from the former in that: (1) the apex of the two lateral processes at the base of the shaft deviates from the shaft; (2) the apex of the pygofer of the abdominal processes is unbranched (Zhang & Qin, 2004a).

#### Distribution.

China (Yunnan).

## Supplementary Material

XML Treatment for Singulus

XML Treatment for Singulus furcatus

XML Treatment for Velu

XML Treatment for Velu claustrum

## ﻿Additional information

### Author ORCIDs

Qing-Ping Yao https://orcid.org/0009-0004-3910-0751

Yan Ding https://orcid.org/0009-0005-3314-040X

Mao-Fa Yang https://orcid.org/0000-0002-5523-6825

Xiao-Fei Yu https://orcid.org/0000-0002-1362-9524

### Data availability

All of the data that support the findings of this study are available in the main text.
